# The Potential of Hydrogel Preparations Containing Plant Materials in Supporting the Treatment of Vaginal and Vulvar Infections—Current State of Knowledge

**DOI:** 10.3390/polym17040470

**Published:** 2025-02-11

**Authors:** Aneta Ostróżka-Cieślik, Monika Michalak, Tomasz Bryś, Marek Kudła

**Affiliations:** 1 Department of Pharmaceutical Technology, Faculty of Pharmaceutical Sciences in Sosnowiec, Medical University of Silesia,41-200 Sosnowiec, Poland; 2 Department of Pharmaceutical Sciences, Medical College, Jan Kochanowski University, 25-317 Kielce, Poland; mmichalak@ujk.edu.pl; 3 Clinical Department of Perinatology and Oncological Gynaecology, Medical University of Silesia, 41-200 Sosnowiec, Poland; tomasz.brys@sum.edu.pl (T.B.); mkudla@sum.edu.pl (M.K.)

**Keywords:** hydrogel, plant, bacterial vaginosis, vulvovaginal candidiasis

## Abstract

Vaginal hydrogels are a modern alternative to solid (tablets, globules) and other semi-solid forms of medication (ointments, creams) in the control of pathogenic microorganisms in diseases of the female reproductive tract. This review aims to summarize the current state of knowledge regarding the efficacy of hydrogels containing plant materials in the treatment of vaginal and vulvar infections. New therapies are essential to address the growing antimicrobial resistance crisis. Google Scholar, Web of Science, Cochrane, and Medline (PubMed) databases were searched. Twenty-five studies were included in the review, including basic, pre-clinical, and clinical studies. The results obtained confirmed the therapeutic potential of plant raw materials embedded in the polymer matrix of hydrogels. However, due to the small number of clinical trials conducted, further research in this area is needed.

## 1. Introduction

Antimicrobial drug resistance is one of the biggest problems in modern medicine. Limited research into new antibiotics/therapies and the overuse of currently available ones on the market have made antibiotic resistance a serious health threat. The World Health Organization (WHO) has identified it as one of the top 10 global public health threats. The European Centre for Disease Prevention and Control (ECDC) estimates 33,000 deaths annually (EU and EEA area) due to antimicrobial resistance. It is projected that this number could rise to 10 million per year by 2050 [1]. The search for compounds with proven therapeutic activity in the face of increasing chemotherapeutic resistance in the treatment of bacterial and fungal infections of the vagina and vulva is therefore imperative. Research is currently being conducted in several directions. One strategy involves the development of combination therapies, using the synergistic action of two or more compounds. Other research is focused on finding new compounds dedicated to the pharmacotherapy of a specific infection. An increasing amount of research is directed toward finding natural therapies using plant materials. It should be borne in mind, however, that treatment based on phytotherapy is for the long term and is mainly considered an adjunctive treatment.

## 2. Materials and Methods

### 2.1. Focused Questions

Do hydrogel preparations containing plant materials show therapeutic potential in the treatment of vaginal and vulvar infections?

### 2.2. Eligibility Criteria

The article provides a narrative review of the literature. Google Scholar, Web of Science, Cochrane, and Medline (PubMed) databases were searched using a combination of MeSH terms, including “hydrogel”, “polymer”, “semi-solid formulation”, “herbal”, “natural compounds”, “*Candida*”, “*Streptococcus*”, “vulvovaginal candidiasis”, “therapeutic treatment”, “vaginal infection”, “antibiofilm activity”, “antifungal”, “bacterial vaginosis”, and “physiochemical properties”. The search procedure has been simplified by using the “AND” operator. Articles published in peer-reviewed journals that included the above-mentioned keywords in their titles and/or abstracts were included. Studies published up to December 2024 were included, with a focus on publications from the last 30 years. Inclusion criteria were original papers with data from basic, preclinical, and clinical studies. Hydrogels based on different types of polymers containing raw materials of plant origin were included. Exclusion criteria were articles that investigated another semi-solid form of drug for vaginal administration containing a plant-based raw material (creams, ointments). Letters to the editor and studies published on websites and in newsletters were not included in the review.

## 3. Causes and Symptoms of Vaginal and Vulvar Infections

One of the most common reasons for patients presenting to gynecologists is a set of symptoms that may indicate vulvovaginitis [2]. These include itching, burning, pain in the urogenital area (also during intercourse), unpleasant vaginal odor, and painful urination [2,3].

A history unsupported by other clinical data suggesting an inflammatory etiology is often insufficient to make a correct diagnosis. Correct management also involves a physical examination and microbiological tests to confirm the initial diagnosis [4]. Finding the cause of the patient’s symptoms is extremely important, as similar symptoms may occur in non-infectious diseases such as lichen sclerosus, lichen planus, psoriasis, vulvodynia, cancer, or allergies to the detergents used for intimate area care [3]. Incorrect diagnosis delays the elimination of bothersome complaints, reducing quality of life and exposing the patient to unnecessary prolonged pharmacotherapy [4].

The microflora of the reproductive tract of women of childbearing age includes many aerobic and anaerobic bacterial species. Although it is accepted that the main strain colonizing the female reproductive tract is *Lactobacillus* spp., it is worth mentioning the somewhat less obvious species, which include *Streptococci type B* (GBS), *Staphylococcus aureus*, *Escherichia coli*, *Klebsiella* spp., and others [3].

The composition of the microflora before puberty in girls and during menopause in women is poorer in lactic acid bacilli, among others, largely resembling the skin microflora. This is related to significantly lower concentrations of estrogen and glycogen, two key factors required for the colonization of the reproductive tract by *Lactobacillus* spp. All components are reflected in the fact that in groups of young girls and older women, the pH of the reproductive tract is closer to neutral (6.0–7.5) in contrast to women of reproductive age (vaginal pH in the range of 3.8–4.2 due to the presence of lactic acid produced by *Lactobacillus* spp.) [3]. For the reasons mentioned above, vulvovaginal infections in these age groups may have a different course. One of the pitfalls concerning the menopausal period may be the symptoms of atrophic vaginitis (part of the urogenital syndrome), which may mistakenly resemble the development of an infection [3].

The development of vulvovaginitis most often has its origin in an imbalance of vaginal biocenosis in favor of a proliferation of pathogenic microorganisms. Such a condition is defined as dysbiosis [3]. It is estimated that every woman will experience an episode of vulvovaginitis at least once in her lifetime [2]. The most common etiology includes bacterial infection (depending on sources, 20–60%; average: 40–50% of cases) [3,5], yeast infection (20–25%) [3], cysticercosis, and viral infection (e.g., HSV—*Herpes Simplex Virus*) [2,3].

Wearing cotton underwear, correct intimate hygiene techniques, the use of condoms (especially during incidental sexual contact), avoiding vaginal irrigation, and regular testing for sexually transmitted diseases play a very important role in the prevention of infection [3,5].

A bacterial vaginal infection called bacterial vaginosis (BV) is a condition in which there is an excessive proliferation of pathological bacterial flora as species such as *Gardnerella vaginalis*, *Mycoplasma hominis*, *Mobiluncus* spp., and others [3]. The overriding symptom is an unpleasant fishy odor emanating from the genital tract along with an increased amount of gray or white-colored discharge. However, the typical inflammation manifested by erythema, fissures, and bleeding of the irritated mucosa is absent. Some patients may not present with any symptoms. The main risk factors for bacterial vaginitis include vaginal irrigation, excessive hygiene of the intimate area, smoking, frequent change in sexual partners, and homosexual contact between women. It is noteworthy that bacterial vaginosis may favor the development of other infections in the genital tract, influencing a local reduction in the immune response and the release of endotoxins that facilitate the initiation of inflammation in the presence of another pathogenic agent [3,5].

A vulvovaginal infection caused by a fungus of the *Candida* species (most commonly *Candida albicans*) is called vaginal candidiasis. It accounts for approximately one-third of all vulvovaginal infections; 70% of patients have experienced at least one episode of candidiasis in their lifetime, while 8% struggle with recurrent infections. Classic symptoms include pruritus, burning, and discharge of thick white vaginal discharge [2,3,6]. At this time, there is a lack of evidence to suggest that a high number of sexual partners may contribute to the development of vulvar candidiasis [6]. However, it is known that pregnancy, diabetes mellitus, obesity, recent antibiotic therapy, and immunocompromised states (long-term intake of corticosteroids and antimetabolites, organ transplant status, HIV infection) predispose to its onset [2,3,6].

*Trichomonas vaginalis* is one of the more common causes of vulvovaginitis, according to sources accounting for 3.2–8.9% of infections [7]. Patients with trichomoniasis report typical symptoms of vulvovaginal infection, including pruritus, discharge with a runny and foamy consistency, yellow or green color, and a specific odor [2,7]. On speculum gynecological examination, *T. vaginalis* infection can be suspected based on a cervix with an appearance resembling the structure of a strawberry [6].

Treatment of the above-described infections follows a variety of regimens. The main antibiotics used to treat BV and cervicitis are metronidazole and tinidazole orally and vaginally [5,7], while candidiasis is treated with drugs from the azole group, also orally or vaginally [2,6]. Due to frequent co-infection with multiple microorganisms, combination preparations of nystatin and nifuratel by the vaginal route are acceptable [8]. Approximately 30% of BV cases are spontaneously curable without pharmacotherapy. An asymptomatic course of BV infection does not require any treatment [5].

## 4. Importance of Plant Raw Materials Used in the Treatment of Gynecological Diseases

Resistance to commonly used pharmacological treatments has prompted increased interest in plant-based alternatives in gynecological practice. Plant extracts are increasingly becoming an object of interest in combating pathogenic microorganisms in diseases of the female reproductive tract. Plants are abundant in biologically active compounds (e.g., flavonoids, phenolic acids, tannins, mucilages, azulenes) or components of essential oils (e.g., thymol, carvacrol, myrcene, α-terpineol, 1,8-cineole, eugenol, α-terpinene, linalool, geraniol, menthone, β-pinene, borneol, sabinene, limonene, p-cymene, thymol, menthol) found in various parts of the plant, including leaves, flowers, aerial parts, fruits, roots, seeds, or shoulders (Table 1). The multidirectional action of plant raw materials allows for their use in the treatment of many gynecological diseases, either as an alternative or natural support in the treatment process [9,10,11].

The available literature indicates the potential use of plant materials for amenorrhea (e.g., *Aloe barbadensis* Mill., *Carica papaya* L., *Tamarindus indica* L., *Sambucus nigra* L., *Petroselinum crispum* (Mill.) Fuss, *Pimpinella anisum* L.), dysmenorrhea (*Acacia leucophloea* L., *Eclipta prostrata* L., *Tephrosia purpurea* Pers., *Valeriana officinalis* L., *Salvia rosmarinus* Spenn.), leucorrhea (*Asparagus racemosus* Wild., *Nelumbo nucifera* Gaertn., *Smilax zeylanica* L., *Clitoria ternatea* L.), menorrhagia (*Cassia occidentalis* L., *Mangifera indica* L., *Desmodium triflorum* L.), gonorrhea (e.g., *Lawsonia inermis* L., *Ficus religiosa* L., *Ceiba pentandra* L.), premenstrual syndromes (PMSs) (e.g., *Vitex agnus-castus* L., *Lycopus europeaus* L., *Cimicifuga racemosa* L., *Crocus sativus* L., *Gingko biloba* L., *Hypericum perforatum* L.), or human papilloma virus (HPV) infection (e.g., *Podophyllum peltatum* L., *Thuja occidentalis* L.) [10,12,14,15,16]. Several plants are also known to be recognized in traditional medicine as having protective and therapeutic properties, including in the context of anti-inflammatory protection (e.g., *Calendula officinalis* L., *Achillea millefolium* L., *Chamomilla recutita* L.) and antimicrobial properties (e.g., *Trigonella foenum-graecum* L., *Azadirachta indica* A. Juss., *Cichorium intybus* L., *Thymus vulgaris* L., *Paeonia suffruticosa* Andr.) [9,12,13,17].

In the treatment of gynecological diseases, raw materials of plant origin can be dosed orally (e.g., decoction, infusion, capsules) or vaginally (e.g., vaginal suppositories, pills, solutions, ointments, creams, gels) [10].

## 5. Potential of Hydrogels in Vaginal Administration

The concept of developing vaginal preparation in the form of a hydrogel stems from the search for alternative forms to the solid and semi-solid drug forms often used. The versatility of tablets, capsules, and vaginal globules is due to their ease of production and greater stability of the drug form. The disadvantage of their use is the risk of uncontrolled removal if applied too shallowly. In addition, vaginal tablets and capsules are pH-dependent, so that API (active pharmaceutical ingredient) absorption can be variable and incomplete in the treatment of various conditions when the pH exceeds 4.5. The physiological conditions of vaginal administration are also a problem. An amount of vaginal secretions of approximately 2 mL may limit the solubility of the active substance [18]. On the other hand, lipophilic ointments and o/w creams do not ensure adhesion to the mucosa and mix poorly with vaginal secretions [19]. An additional advantage of hydrogels is their ease of preparation and low production cost. Current research is being conducted for the development of novel hydrogel drug carriers for gynecological applications.

Hydrogels are matrices containing a loosely cross-linked polymeric structure filled with water. Weak physical hydrogels, in which the polymer chains are mainly linked by hydrogen bonds, are most commonly used for vaginal administration [20]. Common polymers used are Carbopol, polyvinyl alcohol, poloxamer (Pluronic), cellulose derivatives (hydroxypropyl methylcellulose, methylcellulose, hydroxypropyl cellulose, hydroxyethyl cellulose, carboxymethylcellulose), polyethylene glycol, xanthan gum, chitosan, sodium alginate, hyaluronic acid, and others [21,22,23]. It is required that the formulated preparation has the ability of mucoadhesion to the vaginal mucosa, which increases its contact time with the vaginal surface and the bioavailability of the therapeutic substance. The gynecological hydrogel should be easy to apply and non-allergenic and non-irritating to the mucosa. It is important that it has the appropriate rheological and texture properties to allow for optimal spreading of the preparation in the vagina [24]. The challenge is to develop the technology to incorporate the therapeutic substance into the hydrogel matrix, ensuring its prolonged release profile. The introduction of the API as a solution/suspension into the system affects the cross-linking process and the properties of the resulting hydrogel material. Too large a volume of the drug formulation may interfere with the preparation of the hydrogel preparation, while too small a volume may affect its therapeutic properties. A problem that must also be taken into account is the varying vaginal environment during a woman’s life (volume, composition, and pH of vaginal fluids). The pH changes with age, with the phase of the menstrual cycle, with estrogen levels, and with changes in the composition of cervical mucus [25]. Maintaining adequate vaginal pH is one of the most important factors for the effective action of drugs.

The development of pH- or temperature-sensitive gel matrices is one of the current research directions. Under the influence of these factors, the liquid form, i.e., a sol, is transformed into a semi-solid hydrogel form at the application site. The most commonly used so-called smart polymers for the preparation of gels are Carbopol, poloxamer 407, chitosan, alginate, and others. To prolong the API release time from an in situ gynecological gel, the formulation is often supplemented with an additional polymer to increase the viscosity of the system [26]. Other work focuses on the technology to prepare a hydrogel matrix that undergoes mucoadhesion over several days and increases its viscosity when interacting with the vaginal mucosa [27,28]. Attempts are also being made to incorporate multi-compartmental forms of the drug into the hydrogel formulation. The most popular modification is hydrogel matrices containing liposomes [29]. Gels with microspheres or gelled emulsions are also being developed [30].

## 6. Review of Studies on Vaginal Hydrogel Preparations Containing Plant Materials

Table 2 describes the raw materials of plant origin present in the formulation of the hydrogel preparations for vaginal administration discussed in this paper.

By analyzing Table 2, it can be concluded that many chemical compounds have been isolated from plant raw materials, which translates into their diverse mechanisms of action (e.g., antiseptic, anti-bacterial, antifungal, anti-inflammatory, antioxidant, antiviral effects) and, consequently, the possibility of therapeutic activity.

**Figure 1:**
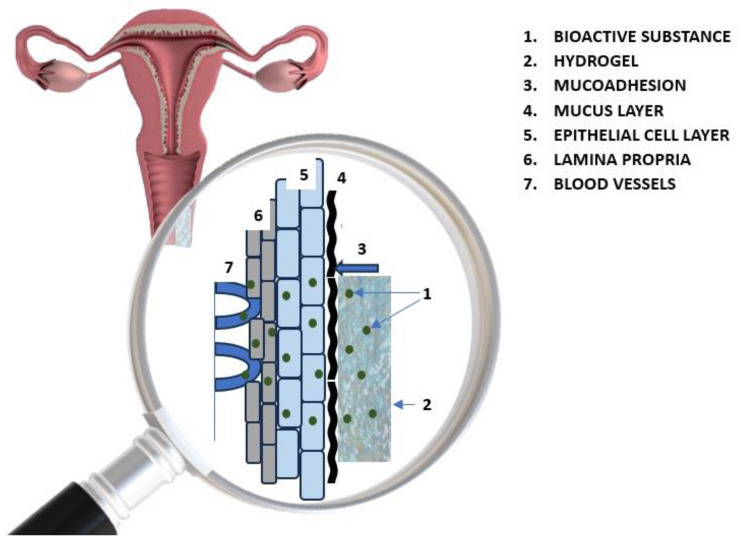
Distribution and diffusion of bioactive substances from the hydrogel after vaginal application.

The content of a polymer matrix can be examined by several methods, most commonly by the incorporation of lyophilized hydrogels into an API solution, homogeneous mixing of bioactive compounds with the polymer in the base precursor, and by covalent binding of active compounds to macromers before the gelation process. Incorporating bioactive compounds into the hydrogel has been found to stabilize bioactive interactions [70]. The chemical affinity of bioactive compounds to the polymer/polymers of the hydrogel matrix (having a large number of domains or hydrophilic groups) is dependent on the water solubility of the plant materials and intermolecular interactions with the functional groups of the polymer hydrogels, i.e., hydrogen bonding interactions with functional groups of the polymer chain such as -OH, -NH_2_, solvophobic effects, and π-π-type interactions between phenolic rings in the plant material [71]. The release of the therapeutic substance from the hydrogel can occur in a controlled manner by diffusion, swelling, or chemical reactions directly into the area requiring treatment. An important determinant of the controlled release of the API is the hydrogel’s degradation rate. Polymeric matrices characterized by a less compact structure and higher water content degrade more rapidly compared to hydrogels with a higher cross-linking density due to the increased mobility of the polymer chains [72]. Figure 1 shows the distribution and diffusion of bioactive substances from the hydrogel after vaginal application.

Table 3 summarizes the current state of knowledge on the efficacy of hydrogels containing plant ingredients in the treatment of vaginal infections.

The therapeutic potential of hydrogels containing raw materials of plant origin is discussed, taking into account the regions of the world where the plant is found.

### 6.1. Basic and Pre-Clinical Research

#### 6.1.1. Global Coverage

*Trigonella foenum-graecum* is native to Asia and Eastern Europe. It is now widely cultivated worldwide [98]. *Cichorium intybus* is cultivated in Europe, India, South Africa, and Chile [99]. *Curcuma longa* is found in India, China, Malaysia, northern Australia, Thailand, and Indonesia [100]. *Azadirachta indica* is a plant native to India [101]. Chopra et al. [74] analyzed polyherbal gels based on *Trigonella foenum-graecum*, *Azadirachta indica*, *Cichorium intybus*, and *Curcuma longa* (NAC) for vaginal drug delivery. A novel vaginal drug delivery system for the topical treatment of aerobic vaginitis was evaluated for its rheological and mucoadhesive properties. Three types of structurally similar and cross-linked acrylic acid polymers. namely Carbopol 934P, Carbopol 974P, and Noveon AA-1 (polycarbophil), in combination with honey and aerosol (fumed silica), were used in this study. The NAC content was 250 mg. A three-level Box–Behnken plan was used to optimize the gel formulation for the highest gel strength and mucoadhesion. The analyzed formulations showed characteristics of viscoelastic solids (G′ > G″). In the in vitro release study, NAC was found to be released from the formulation rapidly within the first hour (according to the zero-order model), while it was released in a prolonged manner over the following 23 h (according to the Korsmeyer-Peppas model). Stability analysis was performed according to ICH guidelines, and the formulations were found to be stable at 6 months. No degradation of NAC and no changes in release profiles were observed in vitro (ANOVA, *p* > 0.05).

Black cumin is native to a vast region of the eastern Mediterranean, northern Africa, the Indian subcontinent, and Southwest Asia, while the olive tree is traditionally found in the Mediterranean basin [102,103]. Sangi et al. [76] attempted to develop a mucoadhesive vaginal gel with microspheres containing *Nigella sativa* and *Olea europaea* oil. Microspheres prepared by the spray drying method were mixed with the carrier using an electric stirrer (50 rpm, 10 min) with a concentration of microspheres in 10% gel. This paper puts an emphasis on the surface morphology of the microspheres, as well as storage stability, in vitro release studies, and the rheological evaluation of gels based on 1% Carbopol 974P NF. The study revealed that the surface morphology of microspheres with black cumin and olive oil observed by a scanning electron microscope were shriveled. The preliminary rheological measurements showed that Carbopol 974P NF is a good matrix for preparing gels for vaginal use due to its good bioadhesive properties. The in vitro release study indicated that the *N. sativa* and *O. europaea* oil microspheres gel had a well-controlled release efficacy.

*Thymbra capitata*, a perennial and ornamental shrub, is an endemic plant to the Mediterranean region [104]. Palmeira-de-Oliveira et al.’s [77] study proposes a novel approach for the treatment of vulvovaginal candidiasis using TCCH hydrogel—*T. capitata* essential oil incorporated in low-molecular-weight chitosan solubilized in lactic acid. The volatile oil used in this study was obtained by hydrodistillation from the air-dried aerial parts of plants. The TCCH hydrogel was assessed for anti-*Candida* activity, including distinct *Candida strains*, such as *C. albicans*, *C. glabrata*, *C. tropicalis*, *C. krusei*, *C. parapsilosis*, and *C. guilliermondii*. It has been shown that the minimal concentration of the pharmaceutical formula containing Mediterranean thyme oil and chitosan with a pH consistent with the vaginal environment has a fungicidal effect on all tested *Candida* strains, both in neutral and acidic pH similar to the vaginal milieu. Confocal microscopy showed that the hydrogel interacts with the yeast cell surface without invasion of the cell. Furthermore, the final product was shown to have a strong effect on the biomass and metabolism of pre-formed *Candida* biofilms, and the antibiofilm effect of the TCCH hydrogel was dose dependent.

*Thymus vulgaris* is a perennial flowering plant found in Europe, Asia, the Mediterranean region, southeastern Italy, and northwestern Africa. About 90% of the world market for thyme oil is produced in Spain [105]. *Vitis vinifera* belongs to the *Vitaceae* family and is cultivated in Asia, North America, Europe, and regions in the subtropical, Mediterranean, and continental–moderate climate belts [106]. *Opuntia ficus-indica* belongs to the cactus family (genus *Opuntia*). The plant is native to Mexico and has become widespread in areas of Central and South America, Australia, the Mediterranean basin, and South Africa [107]. Moraru et al. [89] developed a hydrogel containing thyme essential oil and hydroglyceroalcoholic extracts of *Vitis vinifera* and *Opuntia ficus-indica* powder to help restore and/or maintain the natural vaginal microbiota and maintain its normal homeostasis. The authors investigated the structural and functional properties of two formulations of Kombucha BNC-PX and a hydrogel based on Kombucha BNC (bacterial nanocellulose), CS (chitosan), and PX (poloxamer 407) into which plant extracts/raw materials (thyme essential oil 0.5% *v*/*v*, hydroglyceroalcoholic extracts of *Vitis vinifera* 0.5% *v*/*v*, *Opuntia ficus-indica* powder 0.1% *w*/*v*) were incorporated. Fourier transform infrared spectroscopy (FTIR) and X-ray diffraction (XRD) studies verified daily PX in H1 and H2 in BNC-PX hydrogels, while H3 was in ternary CS packs. In the rheological component, the binary hydrogels push out thixotropy phenomena, while the ternary hydrogels fulfill rheopectic properties. The adhesion energies controlled by BNC-PX and BNC-PX-CS are 1.2 J/m^2^ and 9.1 J/m^2^, respectively. The formulations developed exhibited antimicrobial, antibiofilm, and a high degree of biocompatibility. The hydrogels promoted cell proliferation and promoted the growth of lactic acid bacilli. The test between the analyzed samples (assessed by a one-way ANOVA test) was found to be statistically significant at α = 0.05.

das Neves et al. [75] assessed the possible use of a polycarbophil-based gel containing thyme essential oil (carvacrol type) against *Candida* species, including *C. albicans* H37, *C. albicans* ATCC 10231, *C. glabrata* H16, and *C. krusei* H9. In addition to thyme oil (antifungal agent), polycarbophil (gelling agent and mucoadhesive), triacetin (fixative), lactic acid 90% (acidifier), hydrochloric acid (acidifier and solvent), propylene glycol (moisturizer and solvent), and triethanolamine (neutralizing agent) were used in the formulation of the gel. It has been shown that thyme essential oil possesses promising activity against species commonly involved in vulvovaginal candidiasis and gels with *T. vulgaris* volatile oil are a potent antifungal product. The MICs of both gels containing thyme essential oil as well as *T. vulgaris* essential oils determined at pH 5.5 or pH 7.0 were 0.32 mg/mL. Analyzing the results of the study, the authors concluded that the amount of oil used in the gel (1% *w*/*w*) was sufficient to achieve significant amounts of this substance in the vagina to be effective in the treatment of vaginal infections caused by *Candida* spp.

#### 6.1.2. South America

*Syngonanthus nitens* is found only in South America [108]. dos Santos Ramos et al. [78] describe an interesting solution for the use of nanotechnology for drug delivery systems with mucoadhesive properties. The authors, based on previous knowledge regarding the antifungal properties of the species *Syngonanthus nitens* (Eriocaulaceae), evaluated the potential of a methanolic extract of scapes loaded in a precursor mucoadhesive liquid crystal system for the prevention of vaginal infections caused by *Candida krusei* (ATCC 6258). The formulation developed contained oleic acid as the oil phase, a polymeric dispersion containing synthetic polymers 2.5% Carbopol 974P and 2.5% polycarbophil as the aqueous phase, and PPG-5-CETETH-20 (polyoxypropylene (5) polyoxyethylene (20) cetyl alcohol) as the surfactant. The assessment of the prepared formulations by polarized light microscopy and rheology demonstrated isotropy. Nevertheless, the incorporation of 100% artificial vaginal mucus resulted in a more viscous and anisotropic solution. Moreover, the dilution of the liquid crystal compositions in the artificial vaginal mucus resulted in a notable enhancement in the mucoadhesion parameters. This allowed for a more robust interaction between the preparation and the vaginal mucosa, leading to prolonged retention within the vaginal environment. The antifungal assessments demonstrated that the golden grass extract exhibited activity against all strains examined, with an MIC range of 125 to 62.5 μg/mL. The incorporation of the plant extract into the formulation developed resulted in enhanced activity of *S. nitens* (decreased MIC). Furthermore, the time kill assays indicated that the tested extract was capable of controlling growth, suggesting a fungistatic mechanism. The results of the in vivo prophylactic test conducted on Wistar female rats demonstrated the efficacy of the extract loaded into the mucoadhesive liquid crystal hydrogel in preventing a vaginal infection caused by *C. krusei*, the etiological agent of vulvovaginal candidiasis. To summarize, the authors found that the incorporation of the golden grass extract into polycarbophil/Carbopol 974P-based liquid crystal hydrogel increased its antifungal activity. The formulation can be used as a prophylactic agent for vulvovaginal candidiasis.

The aim of further research of dos Santos Ramos et al. [79] was to broaden the knowledge about the methanolic extract of *Syngonanthus nitens* scapes loaded into a liquid crystal precursor system as a potential agent for the treatment of vulvovaginal candidiasis caused by *Candida albicans*. To prepare the liquid crystal precursor mucoadhesive system, oleic acid was used as the oil phase, while the water phase consisted of a 5% polymeric dispersion synthesized from two polymers, namely 0.5% polycarbophil and 0.5% Carbopol C974P. The surfactant in this system was polyoxypropylene (5) polyoxyethylene (20) cetyl alcohol (PPG-5-CETETH-20)—Procetyl. After incorporating the methanol plant extract into a formulation comprising 40% oil phase, 20% water phase, and 40% surfactant, the effective antifungal activity of the system for all strains tested (the standard strain of *C. albicans* (ATCC 10231)) and five clinical strains (CAV1, 2, 3, 4 and 5) with MICs ranging from 31.2 to 62.5 μg/mL was demonstrated. Microscopic observation revealed that the action of extract loaded into a system was superior to when the extract was not loaded. The analysis of the inhibitory profile of hyphae in *C. albicans* demonstrated an absence of filamentous cells within 24 h of exposure to a concentration of 31.2 μg/mL. The *Syngonanthus nitens* extract, which was not introduced into the drug delivery system, did not show effective activity against biofilms, while the extract incorporated into the liquid crystal precursor showed the inhibition of biofilms of all strains tested. In an in vivo study with Wistar female rats, a system with *S. nitens* extract was effective against *C. albicans* ATCC 10231 after 2 days of treatment and was more potent than the extract itself and the standard drug amphotericin B. Apart from antifungal effectiveness, the authors observed a significant increase in the mucoadhesive parameters, when the liquid crystal precursor compositions (the formulation with and without the plant extract) were diluted by the artificial vaginal mucus. The authors showed that an extract-loaded drug delivery nanotechnological system may be a promising tool in the treatment of vulvovaginal candidiasis. Moreover, it was confirmed that the incorporation into the drug delivery system is important for the increase in plant raw material pharmacological parameters.

*Stryphnodendron adstringens* is a native Brazilian plant [109]. Two research teams, Costa et al. [80] and de Freitas et al. [81], confirmed the efficacy of proanthocyanidin polymeric tannins extracted from *Stryphnodendron adstringens* in reducing the biofilm layer formed by fungi of the genus *Candida* spp. Costa et al. [80] prepared a hydrogel based on Carbopol 940 (1%), into which they incorporated a lyophilized aqueous fraction rich in proanthocyanidin polymers (0.2%). The authors confirmed the physical stability of the formulation. The MIC for *C. albicans* was 31.25 μg/mL. de Freitas et al. [81] used hydrogels containing tannin (fraction F2: 2.5 %*w*/*w* or 5% *w*/*w*) and a subfraction from the stem bark of *Stryphnodendron adstringens* (fraction F2.4) to treat mice with an induced vaginal infection with *C. albicans, C. tropicalis*, *C. parapsilosis*, *C. glabrata. C. krusei*, and *C. guilliermondii.* MICs were established for *C. guilliermondii* and *C. glabrata* (planktonic minimum inhibitory concentration/PMIC ≤ 0.48–3.91 μg/mL), as well as for *C. albicans* (PMIC ≤ 0.48–31.25 μg/mL). It was observed that formulations containing F2 and F2.4 showed similar efficacy against *C. parapsilosis*, *C. krusei*, and *C. albicans.* Efficacy against *C. tropicalis* was lower (*p* < 0.05). An in vivo study in BALB/c mice infected with *C. glabrata* and *C. albicans*, on the other hand, confirmed that the antifungal activity of gels containing F2 and F2.4 was greatest against *C. glabrata* strains than *C. albicans* (*p* < 0.05). The authors concluded that proanthocyanidins derived from *Stryphnodendron adstringens* extracts could be considered as a potential line of treatment for *Candida* infections caused by strains resistant to common antimycotics. The authors suggest that F2 and F2.4 are effective in the treatment of candidiasis in mice.

*Copaifera officinalis* grows in tropical regions of South America [110]. A potential therapeutic effect against *Streptococcus agalactiae* is shown by *Copaifera officinalis*, which is a source of oleoresin. Its mechanism of action mainly involves the disintegration of the bacterial cell wall and membrane. Morguette et al. [83]. prepared hydrogels based on Carbopol 940, into which they incorporated copaiba oil 0.5% (*w*/*w*) or 1.0% (*w*/*w*), obtaining, respectively, CARB-CO 0.5 and CARB-CO 1.0. In in vitro tests, copaiba oil was found to inhibit the growth of all GBS (group *B Streptococcus*) strains dose-dependently, including those resistant to erythromycin and clindamycin. MIC (minimal inhibitory concentration) values were 0.03 and 0.06 mg/mL, and MBC (minimal bactericidal concentration) values were 0.06 and 0.12 mg/mL (*p* > 0.05). Importantly, the oil did not inhibit the growth of *Lactobacillus* strains specific to the human microbiota. By contrast, when testing the developed hydrogels with incorporated copaiba oil in a Carbopol matrix, the authors confirmed antimicrobial activity against GBS strains only for CARB-CO 1.0. This formulation was biocompatible with mouse vaginal mucosa BALB/c. Furthermore, it was characterized by sustained release of the *C. officinalis* oleoresin and exhibited viscoelastic properties. The authors suggest that the Carbopol-based hydrogel is an effective carrier of copaiba oil and may be efficient in controlling *S. agalactiae* colonization and infection. The preparation does not affect the growth of Lactobacilli.

*Mitracarpus frigidus* is a shrub native to South America [111]. Campos et al. [85] developed a gynecological gel containing *Mitracarpus frigidus* (aerial parts) methanolic extract (MFM) and evaluated its efficacy in the treatment of vulvovaginal candidiasis (VVC) in a mouse model. The developed MFM–chitosan gel exhibited characteristics of a pseudoplastic fluid in rheological tests, with increasing viscosity and elasticity with increasing MFM extract concentration (2.5% *w*/*w*, 5.0% *w*/*w*, 10.0% *w*/*w*). SEM (scanning electron microscopy) studies confirmed the therapeutic effect of the developed formulation. A reduction in fungal infection was observed within six days of therapy. No inflammatory infiltration or erythrocytes were observed in the vaginal mucosa. The therapeutic efficacy of MFM–chitosan gel (10% *w*/*w*) was 89.43% and was comparable to the improvement after treatment with the reference formulation (Clotrimazole cream, 10 mg/g) (*p* < 0.05). The authors suggest that the hydrogel may be an alternative for the treatment of *C. albicans* VVC. In addition, the MFM–chitosan gel showed no harmful or toxic effects on the vulvovaginal system.

*Commiphora leptophloeos* is a species of plant found in Bolivia and southeastern Brazil [112]. Dantas-Medeiros et al. [87] evaluated the toxicity and therapeutic efficacy of a *C. leptophloeos* extract (4%) in the form of a vaginal hydrogel based on chitosan (1.0%) and poloxamer 407 (18%). They conducted the study in vitro and in *G. mellonella*, an alternative in vivo model. The formulation showed good physicochemical and microbiological stability and adequate rheological and textural parameters. The authors concluded that the developed hydrogel has high antifungal potential compared to *C. leptophloeos* extract alone at all concentrations used in vitro (2172–33.9 μg/mL) and in vivo at 125 mg/kg. The authors suggest that *C. leptophloeos* extract may be a promising pharmaceutical ingredient for the development of vaginal preparation.

*Annona muricata* (Annonaceae) is found in South America, mainly Brazil [113]. Campos et al. [91] investigated the antimicrobial efficacy of an ethanolic extract obtained from the leaves of *A. muricata* (AME) in an in vivo model of rat vulvovaginal candidiasis. The extract was incorporated into a Carbopol hydrogel matrix with AME concentrations of 1.0% (*w*/*w*), 2.5% (*w*/*w*), and 5.0% (*w*/*w*). A rheological study found that the incorporation of AME into the substrate decreased its viscosity and elasticity with increasing extract concentration, with all developed hydrogels being stable (G‘ > G’). An in vivo vaginal fungal load study confirmed that a Carbopol-based hydrogel with 2.5% AME reduced *C. albicans* infection by 79.9% after 6 days of treatment (2.13 log CFU/mL (day 1) vs. 0.45 log CFU/mL (day 6); *p* < 0.005). The control group consisted of rats treated with nystatin at a dose of 25,000 IU/g, which eliminated infection on the last, i.e., day 6 of treatment (2.05 log CFU/mL (day 1) vs. 0.00 log CFU/mL (day 6); *p* < 0.005). The authors suggest that the developed formulation may effectively support the treatment of vulvovaginal candidiasis.

#### 6.1.3. Asia

Curcumin (CUR) is a compound extracted from the rhizomes of *Curcuma longa*, which is found in southern Asia [114]. Alves et al. [82] prepared thermoresponsive hydrogels based on poloxamer 407 (17–20 g) in combination with hydroxypropyl methylcellulose/HPMC K4M (0.3 g) and chitosan (0.3 g), adding them to curcumin (400 µg/mL, 600 µg/mL or 1200 µg/mL). The researchers used a technique called the solid dispersion technique. Easy-to-apply formulations with optimal cohesiveness, compressibility, adhesion, hardness, and mucoadhesion were obtained. Curcumin was released in a controlled manner according to the Hixson–Crowell model. The liquid-to-gel transition (Tsol-gel) occurred at 36 °C. The developed hydrogel could potentially improve the therapeutic activity of curcumin in vaginal administration. Carvalho et al. [92], based on previous studies, obtained a hybrid carrier nanoformulation (NIN) loaded with benzidamine hydrochloride/BNZ (with anti-inflammatory activity) and curcumin (with antifungal activity) using the microfluidics technique. The formulation was incorporated into a thermosensitive hydrogel based on poloxamer 407 and chitosan (HG). The resulting hydrogels were stable, with optimal rheological parameters. In a pharmaceutical availability study, 52.85% of curcumin was released from the CUR@NIN@HG formulation. The release occurred according to the Higuchi model, i.e., according to Fick’s diffusion kinetics. No antifungal activity of CUR was observed in vitro. However, its therapeutic effect was confirmed under in vivo conditions. Mice treated with CUR+BNZ@NIN@HG showed a reduction in spongiosis (*p* = 0.9815) on histopathology, which is associated with a decrease in fungal load. Hybrid nanoparticles loaded with curcumin and benzydamine hydrochloride minimize the toxicity of CUR and BNZ. The authors confirmed the antifungal and anti-inflammatory therapeutic potential of CUR+BNZ@NIN@HG and demonstrated its therapeutic equivalence to a commercial preparation (clotrimazole). During the experiment, the death of one rodent that was treated with free curcumin occurred (day 22 of the study).

*Scutellariae baicalensis* is found in Asia (eastern Russia, adjacent areas of Mongolia and China). The raw material for making extracts is the tap root [115]. Chanaj-Kaczmarek et al. [86] developed six binary formulations containing *S. baicalensis* radix extract, which they incorporated into a chitosan matrix. An amount of 1 g of lyophilized extract contained 178.10 ± 1.90 mg baicalin, 62.93 ± 1.23 mg baicalein, and 25.31 ± 0.19 mg wogonin. The 60% solutions of freeze-dried *S. baicalensis* radix extract were incorporated into a chitosan matrix at weight ratios of 2:1, 1:1, and 1:2. The formulations showed stability under an acidic vaginal environment (according to ICH Q1A(R2). The amount of baicalein and wogonin released from the formulation (2:1) in vitro was 84.60% and 28.41%, respectively, after 24 h of testing. The formulated preparations showed higher antifungal activity compared to the pure extract (*p* < 0.05). The binary system containing the freeze-dried extract of *S. baicalensis* radix with chitosan mixed in a weight ratio of 2:1 showed a particularly beneficial effect on the vaginal microbiome. The most sensitive strain was *C. parapsilosis*. The therapeutic effect of the 2:1 binary system was comparable to that of metronidazole and clindamycin (*p* < 0.05). The authors suggest that a chitosan-based extract of *S. baicalensis* radix may show therapeutic potential in the treatment of vaginal infections, particularly vulvovaginal candidiasis.

*Paeonia suffruticosa* is a species with its natural occurrence in Asia (growing mainly in the mountains of Eastern, Central, and Southwestern China). Other authors believe that this plant is a hybrid; hence, its origin is difficult to determine [116]. Jia et al. [90] investigated the efficacy of a formulation of carboxymethyl chitosan (CMCS) with the leaf extract of *P. suffruticosa* (PLE) in the treatment of vaginitis in a mouse model. The authors confirmed by SEM, XRD, and FTIR the presence of hydrogen bonds between CMCS and PLE of the developed hydrogel and the stability of the structure of the formulation, which increased with increasing PLE concentration (0.5, 1.0, 1.5, and 2 wt% PLE). The hydrogel was characterized by a porous structure with a three-dimensional network. In a pharmaceutical availability study (in vitro), 92% of the initial PLE dose was released after 12 h, while the complete release was observed after 3 days of testing. Measurements using oscillatory rheology confirmed that the elastic modulus G’ was dominant at the measured frequencies, indicating that the formulation exhibited the properties of an elastic solid gel. In the swelling test, the swelling rate of the hydrogel increased with increasing PLE concentration and was 300–400% (pH = 7.4). On the other hand, in the DPPH and ABTS free radical scavenging method in vitro, the antioxidant activity of the hydrogel was confirmed, which also increased with increasing PLE concentration. At the highest PLE concentration, the highest bacteriostatic index (>96%) was also recorded against bacteria causing vaginitis (i.e., *S. aureus*, *B. streptococcus*, *S. epidermidis*, *E. coli*, *C. albicans*, and MRSA). The study confirmed that CMCS-PLE hydrogel effects restore the balance of the vaginal bacterial flora, making it effective in eliminating the bacteria responsible for mixed infectious vaginitis (*p* < 0.0001).

#### 6.1.4. Africa

*Pelargonium graveolens* is native to South Africa [117]. dos Santos et al. [84] investigated the efficacy of *Pelargonium graveolens* essential oils from Egypt, South Africa (two samples), China, Reunion Island (France), Albania, and Brazil in the treatment of *Candida* spp. fungal infections of the vagina and vulva. A hydrogel-thickened nanoemulsion was prepared by mechanical mixing, combining essential oils (10%) with chitosan (2%). It was hypothesized that the mechanism of action of the active substances involves electrostatic interactions between the components of the formulation and the fungal cell membrane and a pro-oxidant effect on the microorganisms. In vitro results showed that the highest efficacy against *Candida* spp. was obtained with one of the samples from South Africa (MIC 128–256 μg/mL). Similar results were obtained with essential oils from France and China. The most sensitive species to essential oils were *C. parapsilosis*, followed by *C. tropicalis*, with MIC values of 128 μg/mL and 256 μg/mL, respectively. Samples from Brazil and South Africa showed little ability to inhibit the growth of *Candida species* with MICs of 512 μg/mL for most isolates and strains. The results obtained were statistically significant at *p* < 0.05.

#### 6.1.5. Australia and Oceania

*Melaleuca alternifolia* (known as tea tree) is a species endemic to the myrtle family found in Australia (southern Queensland and New South Wales) [118]. Hammer et al. [73] investigated tea tree essential oil, as well as intra-vaginal products containing this oil against *Candida* spp. Melaleuca alternifolia is an Australian native plant of the family *Myrtaceae*. From the leaves of this tree, essential oil is extracted by steam distillation, which has long been used as an effective antimicrobial agent [118]. A study by Hammer [73] confirmed that *Candida* spp. (*C. albicans*, *C. glabrata*, *C. parapsilosis*, and other species) are sensitive (at low concentrations ≤ 0.5%) to tea tree essential oil. The authors also tested *M. alternifolia* essential oil-based products (gel 3%, gel 10%, and pessary 10%), confirming their in vitro anticandidal activity and indicating their potential use in vaginal candidiasis therapy. All tested products showed MICs (0.12–0.25%) and MFCs (0.25–0.5%) similar to non-formulated *M. alternifolia* volatile oil (MIC and MFC were ≤0.25%) for *C. albicans* ATCC 10231. However, further clinical trials are needed to confirm the efficacy of tea tree oil and products containing this anti-yeast active ingredient in vivo. Kola-Mustapha et al. [88] evaluated the antifungal efficacy of *M. alternifolia* and *C. flexuosus* against vaginal strains of *Candida albicans*. The carrier of the oils was an emulgel based on xanthine gum (XG) and guar gum (3:2, 2:3, 4:1, 1:4). The dispersing phase was a mixture of Smix (Tween 80/32% and Span 60/68% with coconut oil). The synergistic effects of the essential oils were investigated, and the therapeutic potential of each oil was separately assessed. The minimum inhibitory concentration (MIC) of the essential oils was 50 µL/mL and 25 µL/mL for *M. alternifolia* and *C. flexuosus*, respectively. An analysis of the fractional inhibitory concentration index (FICI) values showed that the combination of the two oils in a ratio of 75:25 (*C. flexuosus*:*M. alternifolia*) gave the highest antifungal activity. A strong synergistic effect of the oils was obtained with FICI (fractional inhibitory concentration index) values of 0.4 and 0.5. The viscosity of the developed emulsion was 8946.3 ± 8.8 mPas, while pH = 3.76–3.78. The formulation remained stable for one month, indicating the compatibility of the essential oils with the emulgel-type base. The authors suggest a high efficacy of the emulgel in inhibiting the growth of *C. albicans*.

### 6.2. Clinical Research

#### Global Coverage

Clinical studies on the efficacy of hydrogels containing plant raw materials have been carried out on plant species with a wide global range. *Myrtus communis* is native to areas of the Mediterranean basin. It is also found in North Africa, Europe, and West Asia. The range of *Berberis vulgaris* includes most of continental Europe, as well as the Caucasus, northwestern Iran, and northern Anatolia. *Thymus vulgaris* is a plant found in Europe, Asia, and northwestern Africa. *Syzygium aromaticum* is mainly found in Indonesia [119,120,121]. Masoudi’s team [93] compared the therapeutic efficacy of gels containing *Berberis vulgaris* 5% and *Myrtus communis* 2% extracts in a metronidazole base versus a gel containing metronidazole 0.75%. By analyzing the package leaflet for metronidazole vaginal gel 0.75%, we confirmed that the hydrogels were formulated on a Carbopol base. The 120 patients were divided into three groups (*Myrtus communis* in metronidazole base/group A; *Berberis vulgaris* in metronidazole base/group B; metronidazole alone/group C; each group had 40 patients). The duration of therapy was 7 days. After this time, the treatment of infection was found to be highly effective in patients in groups A and B compared with the treatment of patients in group C (*p* < 0.001). Furthermore, there was no significant difference between groups A and B (*p* = 0.18). Thirty percent of patients treated with metronidazole gel alone experienced a recurrence of infection within three weeks. No recurrence of bacterial vaginosis was observed in patients who used *Berberis vulgaris* in a metronidazole base and *Myrtus communis* in a metronidazole base. No serious side effects or recurrences were confirmed in treated patients.

The same authors in subsequent clinical studies [94,95] confirmed the high therapeutic efficacy of *Berberis vulgaris* 5% metronidazole-based vaginal hydrogel (group A/40 patients) and *Myrtus communis* 2% metronidazole-based vaginal hydrogel (group B/40 patients) compared with metronidazole 0.75% vaginal gel (group C/40 patients), *p* < 0.001 and *p* < 0.05, respectively. Extracts were prepared by percolation with 70% ethanol. The authors concluded that the formulations developed increased the efficacy of the treatment of bacterial vaginitis. Patients did not experience serious side effects during treatment. The therapeutic potential of *Berberis vulgaris* was confirmed by the Shabanian team [94]. Patients with bacterial vaginitis were treated vaginally for 5 days with metronidazole hydrogel 0.75% (40 patients/group A) and *Berberis vulgaris* hydrogel 5% (40 patients/group B), respectively. Patients diagnosed with an allergy to barberry were excluded from the study. *Berberis vulgaris* hydrogel 5% showed higher therapeutic efficacy (*p* = 0.001) and improvement in symptoms of irritation, dyspareunia, and dysuria (*p* < 0.05).

Murina et al. [96] evaluated the therapeutic efficacy of a vaginal gel containing extracts of *Thymus vulgaris* and *Eugenia caryophyllus* in the treatment of bacterial vaginitis and vulvovaginal candidiasis. In parallel, two strains of lactobacilli (*Lactobacillus fermentum* LF10 and *Lactobacillus plantarum* LP02) were administered to the patients as extended-release vaginal capsules. The study involved 209 female patients from thirty-eight centers. Of these, 100 were diagnosed with bacterial vaginosis (BV), 82 with vulvovaginal candidiasis (VVC), and 27 had recurrent vulvovaginal candidiasis disease (RVVC). After treatment, microbiological evaluation was performed, which confirmed an adequate status in 80.0% of patients with BV, 62.5% with VVC, and 100.0% with RVVC. Clinical symptoms resolved in 83.9% of cases with BV, 81.6% with VVC, and 63.0% with RVVC (*p* = 0.001). All patients tolerated the prescribed treatment well. The authors point to the lack of randomization, the open-label nature of the study, and the small sample size for RVVC (23 patients) as limitations of the study. Furthermore, they suggest that they could not exclude a placebo effect.

### 6.3. Potential of Antipathogenic Activities of Plants

The wide range of anti-bacterial/bacteriostatic and antifungal activities of plant-derived substances is due to their diverse chemical composition and a broad spectrum of biological activity. These compounds exhibit the ability to penetrate the cell wall and membrane of microorganisms, causing disruption of their integrity. For example, terpenes cause coagulation of the cytoplasm and permeabilization of the bacterial cell membrane. This results in the loss of H+ and K+ ions, the disruption of normal ion pump function, a decrease in membrane potential, and a decrease in intracellular ATP levels [122,123]. The bactericidal action of polyphenols results in the degradation of the pathogen’s cell walls, interacting directly with cytoplasmic membranes (disrupting them), damaging membranes and proteins, disrupting enzymatic reaction mechanisms, and affecting both RNA and DNA synthesis [124,125]. The antifungal activity of natural compounds is in turn due to their ability to damage the integrity and rigidity of the fungal cell wall. Cracking of the cell wall and leakage of cytoplasm may be a consequence of their effect on the action of enzymes involved in cell wall synthesis. Some natural compounds (e.g., thymol, limonene) inhibit cell wall synthases [126].

## 7. Plant Raw Materials—Requirements and Limitations on Their Use

The content of active substances in a plant is influenced by, among other things, geographical location, time of year, and time of day, and it is a species-specific issue. It has been found, for example, that plants harvested in early spring and summer contain the highest alkaloid content. The highest concentrations of essential oils and glycosides are observed in plants harvested in the late morning and early afternoon [127,128,129]. However, it should be emphasized that plant raw materials should come from cultivation or natural sites, and their specific harvesting requirements are defined by the specific monograph of the Polish Pharmacopoeia (FP), the European Pharmacopoeia (Ph. Eur), another country’s Pharmacopoeia, and the European Medicines Agency’s Guidelines. FP/Ph. Eur. distinguishes between three types of extracts, namely extracts standardized for active ingredients (in which limits are defined for the content of the active ingredient/group of ingredients with established therapeutic activity), quantified extracts (containing a precisely defined amount of active markers), and other extracts (defined by a precise description of the manufacturing process and quality specifications) [130,131].

Plant extracts exhibit volatility, high sensitivity to environmental factors, and reduced stability. Hydrogel matrices are effective carriers to minimize these limitations. Plant-based hydrogels are biocompatible and biodegradable with optimal physicochemical parameters [132]. Incorporating plant materials into polymer matrices maintains their biological activity and protects them from degradation. A significant limitation of the technology for preparing this drug form is the plant extract’s variable chemical composition, which depends on the ecological conditions. The qualitative and quantitative composition of the raw material is influenced by external factors, including the age and variety of the plant, the type and quality of the soil, climatic conditions (length of the growing season, sunshine, precipitation), developmental stage, genetic factors, the harvesting method, storage method, flower color, and the extraction method used [132,133]. The plant material intended for pharmaceutical purposes shows variable quality at the stage of its preparation, so it must be standardized for the content of the active ingredient(s) before the hydrogel is prepared. Standardization ensures the adequate efficacy and therapeutic reproducibility of plant drugs.

Herbal extracts are complex mixtures of many active compounds that may interact with other medicines taken by patients. It is suggested that despite their standardization to one/some components, there may be variability in the other compounds present in the raw material. Bioactive components may affect metabolic enzymes, often implicated in drug–drug interactions. Modarai et al. [134] investigated the cytochrome P450 inhibitory effects of coneflower preparation. They chose a standardized, Swiss-registered extract of *Echinacea purpurea* (L.) for evaluation. The authors found that its inhibitory effect varied and depended on the alkylamide content. The CYP3A4 enzyme was the most sensitive to inhibition, while CYP2D6 was the least sensitive. Another study [135] confirmed the CYP3A inhibitory effect of Chinese-specific plants (*Andrographis paniculata*, *Acacia catechu*, *Arctium lappa*, *Bupleurum marginatum*, *Spatholobus suberectus*, *Dysosma versipellis*) due to the presence of polyphenolics. The authors concluded that the raw materials studied may interfere with the metabolism of concurrently administered herbs and drugs, metabolized by CYP3A4. Decreased metabolism of the drug can lead to accumulation in the body and consequently to the development of toxicity. A toxic reaction may also develop as a consequence of the additive effect of the herb and the drug. The study indicates that caution should be exercised when using herbal preparations with other drugs.

Some studies confirm the adverse effects of botanical preparations, which are related to their allergenic effects. The most common allergenic botanicals include German/Roman chamomile (*Chamomilla recutita*, *Chamaemelum nobile*), arnica (*Arnica montana*), Echinacea, marigold (*Calendula officinalis*), elecampane (*Inula helenium*), and tea tree oil [136,137,138]. Some authors suggest caution during the use of botanicals. It is recommended that patients perform a patch test on a small area of skin to check for any hypersensitivity or allergic reaction [139].

## 8. Conclusions

Vaginal hydrogels containing plant material show potential in the treatment of vaginal and vulvar infections. They could be considered for use as part of adjunctive therapy in accordance with pharmaceutical regulations. However, due to the small number of clinical trials conducted to date, it is suggested that research in this area should continue. Further studies should also focus on determining the optimal API dosing regimen. It is also important to study the interaction between the hydrogel carrier and the bioactive compound to optimize release kinetics.

## Figures and Tables

**Table 1:** The most important bioactive compounds and their effects, as well as examples of plant raw materials used in the treatment of gynecological diseases and in the care of intimate areas [9,12,13].

Bioactive Compound	Plant	Plant Part	Activity
Flavonoids	*Solidago virgaurea*	herba	
	*Arctostaphylus uva ursi*	folium	
	*Tropaeolum majus*	flos	
	*Matricaria chamomilla*	flos	antimicrobial
	*Lamium album*	herba, flos	antifungal
Phenolic acids	*Polygonum hydropiper*	herba	antioxidant
	*Salvia officinalis*	herba	anti-inflammatory
	*Scutellaria baicalensis*	radix	
Tannins	*Potentilla anserina*	rhizome	astringent
	*Quercus robur*	cortex	antimicrobial
	*Hamamelis virginiana*	cortex	anti-inflammatory
			reduces the sensation of burning and itching
Mucus	*Althaea officinalis*	folium	covering
	*Malva sylvestris*	flos	softening
	*Verbascum densiflorum*	flos	protective
	*Plantago lanceolata*	semen	anti-inflammatory
	*Linum usitatissimum*	semen	
	*Chamomilla recutita*	anthodium	anti-allergenic
Azulenes	*Achillea millefolium*	herba	bacteriostatic
			anti-bacterial anti-inflammatory disinfectant
	*Melaleuca alternifolia*	folium	
	*Thymus vulgaris*	herba	
	*Rosmarinus officinalis*	herba	
Ingredients of essential oils	*Eucalyptus globulus*	folium	antiseptic
	*Lavandula angustifolia*	flos, herba	anti-inflammatory
	*Mentha piperita*	herba	strong bacterio-
	*Eugenia caryophyllus*	gemma	static properties
	*Pelargonium graveolens*	flos	anti-bacterial
	*Cymbopogon citratus*	folium	antifungal
	*Cuminum cyminum*	semen	
	*Origanum vulgare*	folium	

**Table 2:** Characterization of the plant raw materials contained in the hydrogel preparations in question for vaginal administration.

Plant	Other Name	Family	Part of Plant	Main Constituents	References
*Melaleuca alternifolia* Maiden & Betche	tea tree	Myrtaceae	leaves	- essential oil, including terpinen-4-ol, γ-terpinene, α-terpinene, α-terpinolene, α-terpineol, α-pinene 1,8-cineole, p-cymene, limonene, β-pinene, myrcene, α-felandrene, aromadendrene, viridiflorene, and δ-cadinene	[31,32]
*Trigonella foenum-graecum* L.	fenugreek	Fabaceae	seeds	- mucous substances (galactomannan) -proteins, amino acids (rich in tryptophan and lysine) - alkaloids (trigonelline) - steroidal saponins (diosgenin derivatives, yamogenins, tigogenins, gitogenins, glycosidic combinations: trigofoenosides, foenugraceina, greekine) - lipids (phospholipids, glycolipids), sterols, lecithin, choline - flavonoids (orientin, saponarin, apigenin, luteolin, quercetin, vitexin)	[33]
*Azadirachta indica* A. Juss	neem	Meliaceae	leaves, flowers, fruits, seeds, bark	- isoprenoids (nimbin, nimbidol, nimbidin, salannin, gedunin, azadirachtin) - flavonoids (quercetin, catechin, nimbaflavanone) - fatty acids (arachidic, stearic, oleic acid) - thiols (dipropylsulfide) - acids and their derivatives (e.g., nimbochalcin, nimbocetin, tiglic acid) - coumarins (scopoletin), isocoumarins (margocetin)	[34,35]
*Cichorium intybus* L.	chicory	Asteraceae	seeds, roots, flowers, leaves, whole plant	- phenylpropenoids (esculetin, esculin, cichoriin, umbelliferone, scopoletin) - flavonoids (isoscutellarein, apigenin, luteolin, hyperin, quercetin, quercitrin, kaempferol) - sesquiterpenoids (lactucopicin, intybulide A, cichoriolide A, crepidiaside A, cichoriosides A) - nitrogen compounds (caffeine, theophylline, zeatin, riboside) - organic acids (tartaric acid, oxalic acid, quinic acid, ascorbic acid, citric acid, malic acid, cichoric acid) - polysaccharudes (inulin)	[36]
*Curcuma longa* L.	turmeric	Zingiberaceae	rhizome	- phenolic compounds (curcuminoids: curcumin, demethoxycurcumin, bisdemethoxycurcumin, coumaric acid, calebin A) - polysaccharides (L-arabinose, D-xylose, D-galactose, D-glucose, L-rhamnose) - peptide (turmerin) - carotenoids - volatile oil (curcumenone, dehydrocurdione, arturmerone, bisacumol)	[37,38]
*Thymus vulgaris* L.	common thyme	Lamiaceae	herba	- essential oil (mainly thymol, carvacrol, p-cymene, γ-terpinene, α-pinene, linalool, borneol, 1,8-cyneol, geraniol) - flavonoids (e.g., apigenin, luteolin, naringenin) - phenolic acids (e.g., quinic, rosmarinic, coffee, p-coumaric, syryngic, p-hydroxybenzoenoic, ferulic, gentisic acid)	[39,40,41]
*Nigella sativa* L.	black cumin	Ranunculaceae	seeds	- oil with dominant fatty acids: linoleic, oleic, palmitic - terpenes and terpenoids (thymoquinone and its derivatives: carvacrol, 4-terpineol, α-pinene, thymol, t-anethol, dithymoquinone, p-cymene, longifolene) - alkaloids (nigellicimine, nigellicimine-N-oxide, nigellidine, nigellicine) - polyphenols (caftaric, gentisic, caffeic, chlorogenic, p-coumaric, ferulic, sinapic, cichoric acid, hyperoside, isoquercitrin, rutin, myricetin, fisetin, quercitrin, quercetin, patuletin, luteolin, kaempferol, apigenin)	[42,43,44]
*Olea europaea* L.	olive tree	Oleaceae	leaves	- phenolic acids (e.g., ferulic, gallic, homovanillic, coffee acid) - flavonoids (e.g., apigenin, luteolin, kaempferol, rutin) - glycosides (e.g., oleuropein, verbacoside) - alcohols (e.g., hydroxytyrosol, tyrosol) - oil (mainly oleic acid, palmitic acid, linoleic acid, stearic acid)	[45,46]
*Thymbra capitata* L.	Mediterranean thyme	Lamiaceae	aerial parts	- essential oil (carvacrol, thymol, p-cymene, γ-terpinene, β-caryophyllene, linalool, borneol) - phenolic acids (e.g., rosmarinic, vanilic, caffeic acid) - flavonoids (e.g., taxifolin, quercetin, aromadendrin, eriodictyol, naringenin, ladanein, genkwanin) - terpenoids (e.g., carvacrol, thymol, camphor, α-terpineol)	[47,48,49]
*Syngonanthus nitens* (Bong.) Ruhland	golden grass	Eriocaulaceae	capitula and scapes	- flavones (predominantly luteolin O- and C-glucosides and apigenin O-glucosides) - xanthones (A-1,3,6-trihydroxy-2-methoxyxanthone, B-1,3,6-trihydroxy-2,5-dimethoxyxanthone, 1,5,7-trihydroxy-3, 6-dimethoxyxanthone, 1,3,6,8-tetrahydroxy-2,5-dimethoxyxanthone)	[50,51]
*Stryphnodendron* *adstringens* (Mart.) Coville	barbatimão	Fabaceae	stem bark, roots	- polyphenolic compounds, especially hydrolysable and condensed tannins (gallic acid, catechin, epicatechin, gallocatechin, epigallocatechin, epigallocatechin 3-O-gallate, robinetinidol)	[52]
*Copaifera officinalis* (L.) Kuntze	copaiba balsam	Fabaceae	leaves, stems, roots	- sesquiterpenes (germacrene D), (E)-β-caryophyllene, α-cubebene, β-bourbonene, cis-α-bergamotene, α-calacorene, selina-3,7-(11)-diene, α-gurjunene	[53]
*Pelargonium* *graveolens* L’Hér.	rose geranium	Geraniaceae	leaves, flowers, aerial parts	- essential oil (citronellol, citronellyl formate, γ-eudesmol, isomenthone, geranyl formate, germacerene D, geranyl butanoate) - flavonoids (kaempferol 3-O-rhamnoside-glucoside, isorhamnetin aglycone, quercetin 3-O-glucoside, kaempferol 3,7-di-O-glucoside, quercetin 3-O-pentose and kaempferol 3-O-glucoside, quercetin 3-O-rhamnoside-glucoside, quercetin 3-O-pentoside-glucoside, myrisetin 3-O-glucoside-rhamnoside)	[54,55]
*Mitracarpus frigidus* (Willd.) K.Schum.	girdlepod	Rubiaceae	aerial parts	- kaempferol, kaempferol-O-rutenoside, rutin, quercetin-hexosylpentoside, kaempferol-rhamnosylhexoside, quercetin-pentosylrhamnosylhexoside, chlorogenic and ursolic acid - clarinoside, harounoside - pyranonaphthoquinone psychorubrin - 2-azaanthraquinone	[56,57]
*Scutellariae baicalensis* Georgi.	Chinese skullcap	Lamiaceae	roots	- flavonoids (baicalin, baicalein, wogonoside, wogonin, oroxylin A, oroxylin A-7-glucuronide, apigenin 7-O-glucuronide, skullcapflavone II) - terpenoids, - volatile oils - polysaccharides	[58,59]
*Commiphora leptophloeos* (Mart.) J. B. Gillet	imburana	Burseraceae	stem bark	- phenolic acids (e.g., gallic, chlorogenic, protocatechuic, quinic acid) - O- and C-glycosylated flavonoids - A- and B-type polymeric proanthocyanidins - coumarins - lignans	[60]
*Cymbopogon flexuosus* (Steud.) Wats.	lemongrass	Poaceae	aerial parts	- essential oil, including citral-a, citral-b, citronellol, geraniol, geranyl acetate, limonene, linalol, nerol, piperitone, α-terpineol, thujane, α-bisabolol, isointermedeol, borneol	[61]
*Vitis vinifera* L.	common grape vine	Vitaceae	fruit	- vitamin C and E - carotenoids (lutein, β-carotene) - flavonols (quercetin-3-glucoside, quercetin-3-rutinoside) - flavanols (procyanidin B1, catechin, epicatechin, epigallocatechin) - hydroxycinnamic acid derivatives (caftaric acid, coumaroyl tartaric acid) - anthocyanins (delphinidin-3-glucoside, cyanidin-3-glucoside, petunidin-3-glucoside, peonidin-3-glucoside, malvidin-3-glucoside, malvidin-3-coumaroyl glucoside) - stilbenes (piceid, trans-resveratrol)	[62]
*Opuntia ficus-indica* L. Mill	prickly pear	Cactaceae	fruits	- flavonoids, mainly isorhamnetin glycoside derivatives, kaempferol - glycosyl-rhamnoside, rutin - organic acids (ascorbic, glutaric, malic, succinic, pyruvic, quinic, citric, piscidic acid) - betalains (betacyanins (betanidin-5-O-β-sophoroside, etanidin-5-O-β-glucoside (betanin), isobetanin, gomphrenin I and betanidin)	[63]
*Paeonia suffruticosa* Andr.	woody peony	Paeoniaceae	flowers	- essential oil (geraniol, citronellol, pentadecane, tricosane, pentacosane, 6,9-heptadecadiene, trans-8-heptadecene, germacrene D, trans-β-ocimene) - paeonol, 2-hydroxy-4-methoxy acetophenone	[64,65]
*Annona muricata* L.	soursop, graviola	Annonaceae	fruits, leaves	- alkaloids (annonaine, nornuciferine, asimilobine, isolaureline, anonaine, xylopine) - annonaceous acetogenin (epomusenin, epomurinin, cis-annoreticuin, muricin, annohexocin, annomuricin, muricatocin) - megastigmanes (annoionol, vomifoliol, roseoside, loliolide) - polyphenols (cinnamic and caffeic acid derivative, epicatechine, quercetin 3-O-rutinosid, kaempferol) - minerals (K, Ca, Na, Cu, Fe, Mg) - essential oils (β-pinene, germacrene D, α-pinene, β-elemene)	[66]
*Myrtus communis* L.	myrtle	Myrtaceae	leaves	- essential oil, mainly 1,8-cineole, linalool, eugenol, α-terpineol, γ-terpinene, myrtenyl-acetate, α-pinene, heptyl isobutanoate, geranyl-acetate, α-terpineol, (Z)-caryophyllene, α-humulene	[67]
*Berberis vulgaris* L.	barberry	Berberidaceae	roots, rhizomes, stem, bark	- alkaloid berberine - quercetin 3-O-glucuronide, narirutin, rutin, kaempferol, apigenin, hydroxyferulic acid, piceatannol, lignan, lehmannin, taxifolin 3-O-rhamnoside, gallic acid, galangin, ferulic acid, p-coumaric acid	[68]
*Syzygium aromaticum* L.	clove	Myrtaceae	leaves, buds	- essential oil (eugenol, eugenyl acetate, eugenol, β-caryophyllene, 2-heptanone, α-humulene, calacorene, humulenol, calamenene) - polyphenols (quercetin, kaempferol, ferulic, caffeic, ellagic, and salicylic acid)	[69]

**Table 3:** Research on the therapeutic efficacy of hydrogels containing plant materials.

Author, Year of Publication	Hydrogel Composition	Plant Source	Experimental Model	Species Tested	Infection Type	Achieved Effects
**Basic and preclinical studies**						
Hammer et al., 1998 [73]	ns	*Melaleuca alternifolia* essential oil	in vitro strain of *C. albicans* (ATCC 10231)	*C. albicans*	vaginal candidiasis	tea tree oil (0.5% *v*/*v*) maintained in vitro efficacy tea tree oil had an MIC of 0.25% (*w*/*v*)—gel 3% tea tree oil had an MIC of 0.12% (*w*/*v*)—gel 10%
Chopra et al., 2007 [74]	Carbopol 934P, Carbopol 974P, Noveon AA-1 (polycarbophil) -based hydrogel	NAC extracted from *Trigonella foenum-graecum*, *Azadirachta indica*, *Cichorium intybus*, *Curcuma longa*	basic studies: rheological measurements, in vitro release, stability studies	ns	aerobic vaginitis	mucoadhesive polyherbal vaginal gels of a NAC using Carbopol 974P were more consistent and provided better regulation of G′ and MDF over a stability period of 6 months than Carbopol 934P and Noveon AA-1 gels
das Neves et al., 2009 [75]	Polycarbophil-based gel	*Thymus vulgaris* essential oil	in vitro four strains of *Candida* spp.	*C. albicans*, *C. glabrata*, *C. krusei*	vulvovaginal candidosis	antifungal activity against *Candida* spp. of gel containing thyme essential oil (1%, *w*/*w*)
Sangi et al., 2011 [76]	Carbopol 974P- based hydrogel	Microspheres containing *Nigella sativa* and *Olea europaea* oil	basic studies: in vitro release, stability studies	ns	vaginal infections	in vitro release studies showed 98.34% releases for NSSOMgel during storage formulation, the surface morphology and content of *Nigella sativa* and olive oil had no notable changes
Palmeira-de-Oliveira et al., 2013 [77]	Chitosan-based hydrogel	*Thymbra capitata* essential oil	*Candida* isolates from mucocutaneous infections and *Candida* biofilms	*C. albicans*, *C. krusei*, *C. glabrata*, *C. parapsilosis*, *C. tropicalis*, *C. guilliermondii*	vulvovaginal candidiasis	increased activity by chitosan hydrogel associated with *T. capitata* essential oil exhibits a fungicidal effect against all tested *Candida* strains hydrogel formulation disrupts the *Candida* biofilm in a dose-dependent manner; the MIC value of 125 mg/mL of TCCH has a potent effect on the biomass and metabolism of *Candida* biofilms
dos Santos Ramos et al., 2015 [78]	Polycarbophil/Carbopol 974P- based liquid-crystal hydrogel	Methanolic extract of *Syngonanthus nitens* scapes	in vitro strain of *C. krusei* (ATCC 6258) and three clinical strains from the vaginal region (CKV1, CKV2, CKV3), in vivo female Wistar rats (n = 24)	*C. krusei*	vulvovaginal candidiasis	incorporation of the extract of *S. nitens* into the mucoadhesive liquid crystal hydrogel increased its antifungal activity formulation with loaded extract was able to prevent the development of infection following 10 days of administration
dos Santos Ramos et al., 2016 [79]	Polycarbophil/Carbopol 974P- based liquid crystal hydrogel	Methanolic extract of *Syngonanthus nitens* scapes	in vitro strain of *C. albicans* (ATCC 10231) and clinical strains (CAV1, CAV2, CAV3, CAV4, CAV5), in vivo female Wistar rats (n = 78)	*C. albicans*	vulvovaginal candidiasis	formulations had strong mucoadhesion, with MIC values in the range of 31.2–62.5 µg/mL microscopic observation revealed an absence of filamentous cells 24 h of exposure to a concentration of 31.2 μg/mL formulation was effective in vivo in the treatment of infection after only 2 days of treatment
Costa et al., 2018 [80]	Carbopol-940-based hydrogel	Extract of *Stryphnode-ndron* *adstringens*	strains of *C. albicans* (ATCC 10230)	*C. albicans*	vaginal candidiasis	hydrogel shows adequate stability MIC of gel was equivalent to 31.25 μg/mL of polymer-rich fraction
de Freitas et al., 2018 [81]	Carbopol-based hydrogel	Tannin fractions from *Stryphnode-ndron* *adstringens*	strains of *C. albicans* *C. glabrata*, in vivo female BALB/c mice	*C. albicans*, *C. glabrata*	vaginal candidiasis	gel containing tannins efficiently controlled vaginal infection by *C. albicans* and *C. glabrata* in mice no toxicity of the gel to vaginal tissue
Alves et al., 2018 [82]	Poloxamer 407, chitosan, and HPMC K4M-based hydrogel	Curcumin	HeLa cells	not specified	vaginal mucositis, bacterial infection, HPV infection	hydrogels have potential for vaginal drug delivery 52% of CUR released over 180 min (Hixson–Crowell model) hydrogel containing CUR can be used as an antioxidant agent in the treatment of diseases that result from oxidative stress
Morguette et al., 2019 [83]	Carbopol-940-based hydrogel	Oleoresin from *Copaifera* *officinalis*	female BALB/c mice	*S. agalactiae*	bacterial vaginosis	the hydrogel showed good biocompatibility with the murine vaginal mucosa oleoresin from *C. officinalis* incorporated into a hydrogel (1.0%—CARB-CO 1.0) exhibits bactericidal and antibiofilm activity toward *S. agalactiae*
dos Santos et al., 2020 [84]	Chitosan-based hydrogel-thickened nanoemulsion	*Pelargonium* *graveolens* essential oil	HET-CAM test	*C. albicans*, *C. krusei*, *C. tropicalis*, *C. parapsilosis*, *C. glabrata*	vaginal candidiasis	mucoadhesive properties the chitosan hydrogel expressed antifungal activity against *Candida* and was found superior to the nanoemulsion containing essential oil 64 times reduction in MIC was obtained for *Candida*
Campos et al., 2020 [85]	Chitosan-based gel	Methanolic extract of *Mitracarpus frigidus*	female Wistar rats (n = 36)	*C. albicans*	vulvovaginal candidiasis	epithelial lesions in the vulvovaginal mucosa (cell death and vacuolization) were less noticeable there were no inflammatory infiltrating cells and erythrocytes in the vaginal mucosa
Chanaj-Kaczmarek et al., 2022 [86]	Chitosan-based gel	*Scutellariae baicalensis* extract	in vitro strain of *G. vaginalis* (ATCC 14018), *S. agalactiae* (ATCC BAA611), *S. aureus* (ATCC 25923), *E. coli* (ATCC 25922), and yeast-like fungi: *C. albicans* ATCC 3153, *C. parapsilosis* ATCC 2195, *C. krusei* ATCC 573)	*G. vaginalis*, *S. agalactiae*, *S. aureus,* *E. coli,* *C. albicans*, *C. parapsilosis,* *C. krusei*	vulvovaginal candidiasis	combination of *S. baicalensis*-lyophilized extract with chitosan allowed the systems with a high antifungal activity combination of *S. baicalensis* extract and chitosan significantly increased the antimicrobial activity against *Candida* species
Dantas-Medeiros et al., 2023 [87]	Chitosan and poloxamer 407 based hydrogel	Extract of *Commiphora leptophloeos*	in vitro uterine/endometrial epithelial cells (HEC-1A); in vivo model of *Galleria mellonella*	*C. albicans*	vulvovaginal candidiasis	hydrogel is biocompatible with the vaginal environment hydrogel increased the antifungal effect and limited the toxicity of *Commiphora leptophloeos* extract higher antifungal potential (vs. free extract) at all tested concentrations in vitro (2172–33.9 μg/mL) and in vivo at a dose of 125 mg/kg
Kola-Mustapha et al., 2023 [88]	Xanthan gum-based emulgel	Essential oils of *Melaleuca alternifolia* and *Cymbopogon flexuosus*	basic studies: viscosity, compatibility study, stability test; vagina women’s swabs (n = 47)	*C. albicans*	vulvovaginal candidiasis	MIC of *Melaleuca alternifolia* was seen to be 50 µL/mL MIC of *Cymbopogon flexuous* was seen to be 25 µL/mL the best antifungal activity was seen when essential oils were combined at the ratio of 75:25 (*C. flexuosus*:*M. alternifolia*)
Moraru et al., 2023 [89]	NDBNC-PX hydrogel NDBNC-PX-CS hydrogel (CS, chitosan; NDBNC, never-dried bacterial nanocellulose; PX, poloxamer 407)	*Thymus vulgaris* essential oil, hydro-glycero-alcoholic extract of *Vitis vinifera*, *Opuntia ficus-indica* powder	basic studies: FTIR spectroscopy, XRD, TEM, rheology; cell viability assay: the NCTC cell line (clone 929); antimicrobial and antibiofilm activity	*E. coli* *C. albicans*	vulvovaginal candidiasis	hydrogels exhibited a high degree of biocompatibility, with the potential to support cell proliferation and promote lactobacilli growth significant antimicrobial and antibiofilm activity adhesion energies: 1.2 J/m^2^ and 9.1 J/m^2^ gel transition temperatures: 18–22 °C (for the binary hydrogels) with thixotropic
Jia et al., 2024 [90]	Carboxymethyl chitosan hydrogel	*Paeonia* *suffruticosa* extract	female BALB/c mice (n = 40)	*C. albicans*, *S. aureus*, *S. epidermidis*, *E. coli*, *B. streptococcus*, MRSA	aerobic vaginitis, vulvovaginitis	hydrogel containing 2 wt% of PLE: the bacteriostatic rate of each bacterium reached >96% hydrogel containing 2 wt% of PLE restores disturbed vaginal flora to a healthy and stable state under pathological conditions in vivo hydrogel containing 2 wt% of PLE had an anti-bacterial effect on the pathogens of mixed infectious vaginitis
Campos et al., 2024 [91]	Carbopol-based hydrogel	*Annona* *muricata* extract	female Wistar rats (n = 36)	*C. albicans*	vulvovaginal candidiasis	significant reduction in vulvovaginal fungal burden and infection reduction in mucosal inflammation
Carvalho et al., 2024 [92]	Chitosan and poloxamer 407-based hydrogel	Hybrid nanoparticles dual-loaded with curcumin and benzydamine hydrochloride	in vitro strain of *C. albicans* (ATCC 18 804 and FMB-01), in vivo female BALB/c mice (n = 76)	*C. albicans*	vulvovaginal candidiasis	drug loading in the nanoparticle reduced their toxicity the antifungal activity of the formulation against resistant *Candida albicans* strains was found in vivo no antifungal activity was observed in vitro
			**Clinical studies**			
Masoudi et al., 2016a [93]	Carbopol-based hydrogel	*Myrtus communis* 2% in metronidazole vaginal gel 0.75%; *Berberis vulgaris* 5% in metronidazole vaginal gel 0.75%	120 women aged 18–40 years	ns	bacterial vaginosis	groups A and B had a better response than C: *Myrtus communis* 2% in metronidazole; vaginal gel 0.75% (group A); *Berberis vulgaris* 5% in metronidazole vaginal gel 0.75% (group B); metronidazole gel alone (group C) there was no significant difference between A and B groups (*p* = 0.18) the patients in groups A or B did not experience any relapse bacterial vaginosis, but in the C group, 30% of patients experienced relapse during the three-week follow-up treatment with a combination of *Myrtus communis* or *Berberis vulgaris* in the metronidazole base improves the efficacy of bacterial vaginosis therapy
Masoudi et al., 2016b [94]	Carbopol-based hydrogel	*Berberis vulgaris* 5% in metronidazole vaginal gel 0.75%	80 women aged 18–40 years	ns	bacterial vaginosis	*Berberis vulgaris* group had a better response than the metronidazole gel alone group adding *Berberis vulgaris* fruit extract to metronidazole gel improves the efficacy of bacterial vaginosis therapy
Masoudi et al., 2017 [95]	Carbopol-based hydrogel	*Myrtus communis* 2% in metronidazole vaginal gel 0.75%	80 women of 18–40 years old	ns	bacterial vaginosis	the combination of metronidazole and *Myrtus communis had* a higher efficiency adding *Myrtus communis* extract to the metronidazole gel base increases the efficiency of bacterial vaginosis treatment
Murina et al., 2018 [96]	Xanthan gum	Extracts of *Thymus vulgaris* and *Syzygium aromaticum*	209 women (mean age: 35.8 years old)	100 women had BV, 82 had VVC, and 27 had RVVC (recurrent VVC)	bacterial vaginosis and vulvovaginal candidiasis	there was a statistically significant improvement in pruritus, burning, vulvovaginal edema and erythema, dyspareunia, and vaginal secretions in all groups microbiological evaluation was normal in 80.0% of cases with BV, 62.5% of cases with VVC, and 100.0% with RVVC
Shabanian et al., 2019 [97]	ns	Extracts of *Berberis vulgaris*	80 women (mean age: 34 years old)	80 women with BV	bacterial vaginosis	*Berberis vulgaris* gel was more effective than metronidazole gel 0.75% treatment with *Berberis vulgaris* gel prevents the recurrence of the disease

Abbreviations: BALB/c, albino, laboratory-bred strain of the house mouse; BV, bacterial vaginosis; CARB-CO 1.0, hydrogels without Carbopol R 940 and 1% copaiba oil; CMCS, carboxymethyl chitosan; CUR, curcumin; FTIR, Fourier transform infrared; G’, elastic modulus; HET-CAM, Hen’s Egg Test Chorioallantoic Membrane; HPMC K4M, hydroxypropyl methylcellulose K4M; MDF, maximum detachment force; MIC, minimum inhibitory concentration; MRSA, methicillin-resistant *Staphylococcus aureus*; NAC, novel antimicrobial combination; ns, not specified; NSSOMgel, *Nigella sativa* and olive oil microspheres in gel; PLE, *P. suffruticosa* leaf extract; RVVC, recurrent VVC; TCCH, *Thymbra capitata* essential oil and chitosan; TEM, transmission electron microscopy; VVC, vulvovaginal candidiasis; XRD, X-ray diffraction.

## Data Availability

The data presented in this study are available upon request from the corresponding author.
